# Two Distinct Types of Sweat Profile in Healthy Subjects While Exercising at Constant Power Output Measured by a Wearable Sweat Sensor

**DOI:** 10.1038/s41598-019-54202-1

**Published:** 2019-11-29

**Authors:** Dong-Hoon Choi, Grant Kitchen, Ji Soo Kim, Yi Li, Kain Kim, In cheol Jeong, Jane Nguyen, Kerry J. Stewart, Scott L. Zeger, Peter C. Searson

**Affiliations:** 1 Institute for Nanobiotechnology, John Hopkins University, 3400 North Charles Street, Baltimore, Maryland 21218 USA; 2 Department of Biostatistics, Johns Hopkins University, 615 North Wolfe Street, Baltimore, Maryland 21205 USA; 3 Department of Medicine, Johns Hopkins University School of Medicine, 4940 Eastern Ave, Baltimore, Maryland 21224 USA; 4 Department of Materials Science and Engineering, 3400 North Charles Street, Baltimore, Maryland 21218 USA

**Keywords:** Sensors, Biomedical engineering

## Abstract

Wearable sweat sensors have enabled real-time monitoring of sweat profiles (sweat concentration versus time) and could enable monitoring of electrolyte loss during exercise or for individuals working in extreme environments. To assess the feasibility of using a wearable sweat chloride sensor for real-time monitoring of individuals during exercise, we recorded and analyzed the sweat profiles of 50 healthy subjects while spinning at 75 Watts for 1 hour. The measured sweat chloride concentrations were in the range from 2.9–34 mM. The sweat profiles showed two distinct sweat responses: Type 1 (single plateau) and Type 2 (multiple plateaus). Subjects with Type 2 profiles had higher sweat chloride concentration and weight loss, higher maximum heart rate, and larger changes in heart rate and rating of perceived exertion during the trial compared to subjects with Type 1 profiles. To assess the influence of level of effort, we recorded sweat profiles for five subjects at 75 W, 100 W, and 125 W. While all five subjects showed Type 1 sweat profiles at 75 W, four of the subjects had Type 2 profiles at 125 W, showing an increase in sweat chloride with exercise intensity. Finally, we show that sweat profiles along with other physiological parameters can be used to predict fluid loss.

## Introduction

Secretion of fluid by sweat glands in the skin can occur in response to various thermal and chemical stimuli. Sweat contains sodium, potassium, and chloride ions and hence sweating is directly coupled with changes in electrolyte balance in the body. In healthy individuals, the electrolyte and water loss during sweating can lead to a range of conditions, such as hypo- and hypernatremia. Exercise performance is also influenced by electrolyte loss and dehydration^1–6^. Furthermore, working in hot environments with poor hydration has been linked to chronic kidney disease^7,8^. The recent development of wearable sweat sensors^9–18^ provides new opportunities to study fundamental issues associated with electrolyte loss in health and disease, enabling real-time measurement of the dynamic response outside of the laboratory. Previous studies have largely relied on laboratory analysis of samples collected during a trial^19–21^, which precludes real-time analysis. From a health and fitness perspective, wearable sweat sensors provide real time data that could be used to prevent heat-related injury and optimize athletic performance^22–24^.

Sweating is complex thermoregulation process that is dependent on physiological responses including cardiovascular function, skin and core temperature responses, plasma volume, and sweat rate^25–27^. Wearable technologies have the potential to contribute to the understanding of sweating, and to develop tools to predict and prevent heat-related injuries. In previous work we have developed a wearable sweat sensor^9–11^ and have validated sensor performance in studies with healthy subjects and individuals with CF following chemically induced sweating^10^. Here, building on our previous work, we make three key advances. First, to demonstrate the potential for real-time measurement during exercise, we performed on-body trials with 50 healthy subjects while spinning on an exercise bike at 75 W for 60 minutes. To assess the link between the sweat profile and other physiological parameters, we simultaneously monitored heart rate, core temperature, skin temperature, and the rating of perceived exertion (RPE), along with overall weight loss. Second, we classified the 50 subjects into two groups based on their sweat profiles and assessed the relationship between the sweat profiles and other physiological responses. To assess the influence of level of effort on sweat profile, we performed trials with five subjects at 75, 100, and 125 W for 1 hour. Third, using machine learning, we showed how the physiological data measured during exercise can be used to predict risk for dehydration due to excessive fluid loss.

## Results and Discussion

### Wearable sweat chloride sensor

**Figure 1:**
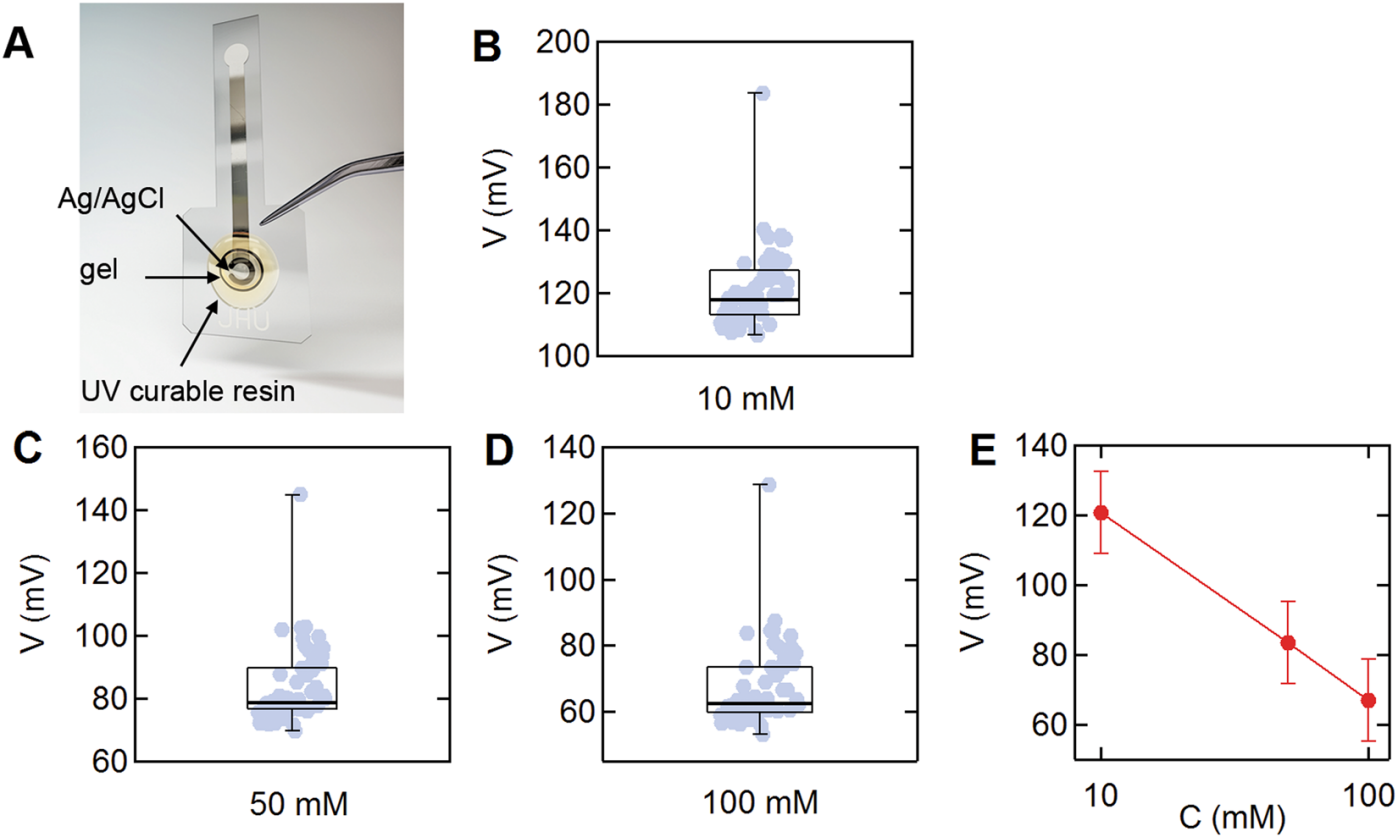
Wearable sweat chloride sensor. (**A**) Photograph of the potentiometric sweat sensor. (**B**–**D**) Distribution of sensor voltages in (**B**) 10 mM, (**C**) 50 mM, and 100 mM NaCl solutions (N = 65). Box and whisker plots display the minimum, first quartile, median, third quartile, and maximum values. (**E**) Average calibration curve obtained from the 65 sensors used in this study.

All trials were performed using a wearable potentiometric sweat chloride sensor developed in our lab (Fig. 1A)^10,11^. We used a total of 65 sensors to record sweat profiles, and all sensors were calibrated prior to each trial. The voltages of 65 devices in 10, 50, and 100 mM of three standard sodium chloride (NaCl) solutions were 120.8 ± 11.8, 83.4 ± 11.8 and 67.1 ± 11.8 mV (mean ± SD) mV, respectively (Fig. 1B–D). The slope and y-intercept of the calibration values obtained from the voltages were −53.6 ± 3.5 (mean ± SD (standard deviation)) mV/decade and 174.4 ± 12.9 (SD) mV, respectively (Fig. 1E). If the average calibration curve was used for the standard measurements, the error in the 10, 50, and 100 mM solutions was 1.0 ± 4.0, 4.7 ± 19.1, and 10.2 ± 38.1 mM (mean ± SD), respectively.

### Variation of the sweat chloride concentration among healthy subjects

**Figure 2:**
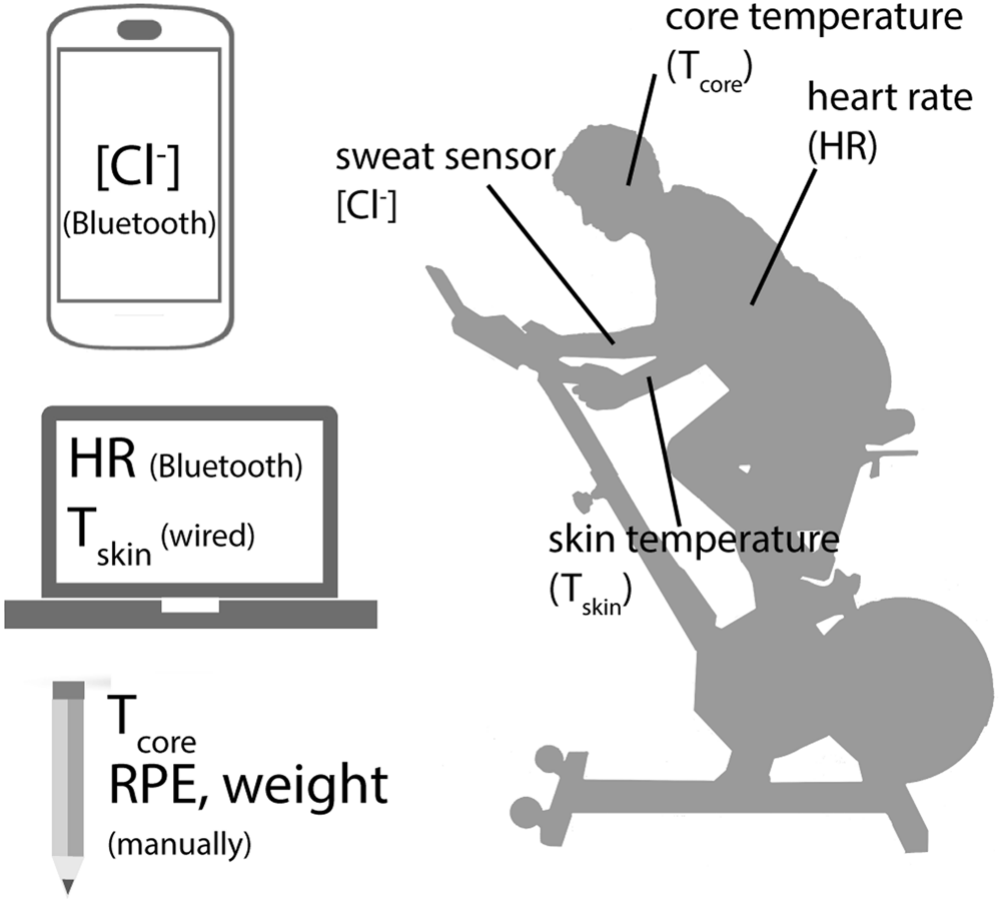
On-body trials to measure sweat profile, heart rate, core and skin temperature and weight loss during exercise. The sweat sensor and heart rate sensor were wirelessly (Bluetooth) connected to a smart phone and a laptop and the data were recorded in real time with a 1 Hz sampling rate. The skin temperature (T_skin_) sensor was wired and connected to a laptop, and it was sampled at 2 Hz. The core temperature (T_core_) and RPE values were manually recorded every 5 minutes. The subjects’ weight was measured before and after the trial.

50 healthy individuals were recruited to spin at a constant power output of 75 W for 60 minutes following a 25 minute warm-up (Fig. S1 in Supplementary Information). Figure 2 shows the setup of the trial in this study. During the trials, the sweat chloride concentration was wirelessly monitored using a Bluetooth transceiver and mobile app with a 1 Hz sampling rate. The heart rate was also wirelessly monitored at a 1 Hz sampling rate, and a thermocouple sensor was used to monitor skin temperature at a 2 Hz sampling rate. The core temperature and RPE were manually recorded every 5 minutes, and the overall weight change was obtained by measuring the subject’s weight before and after the trial.

**Figure 3:**
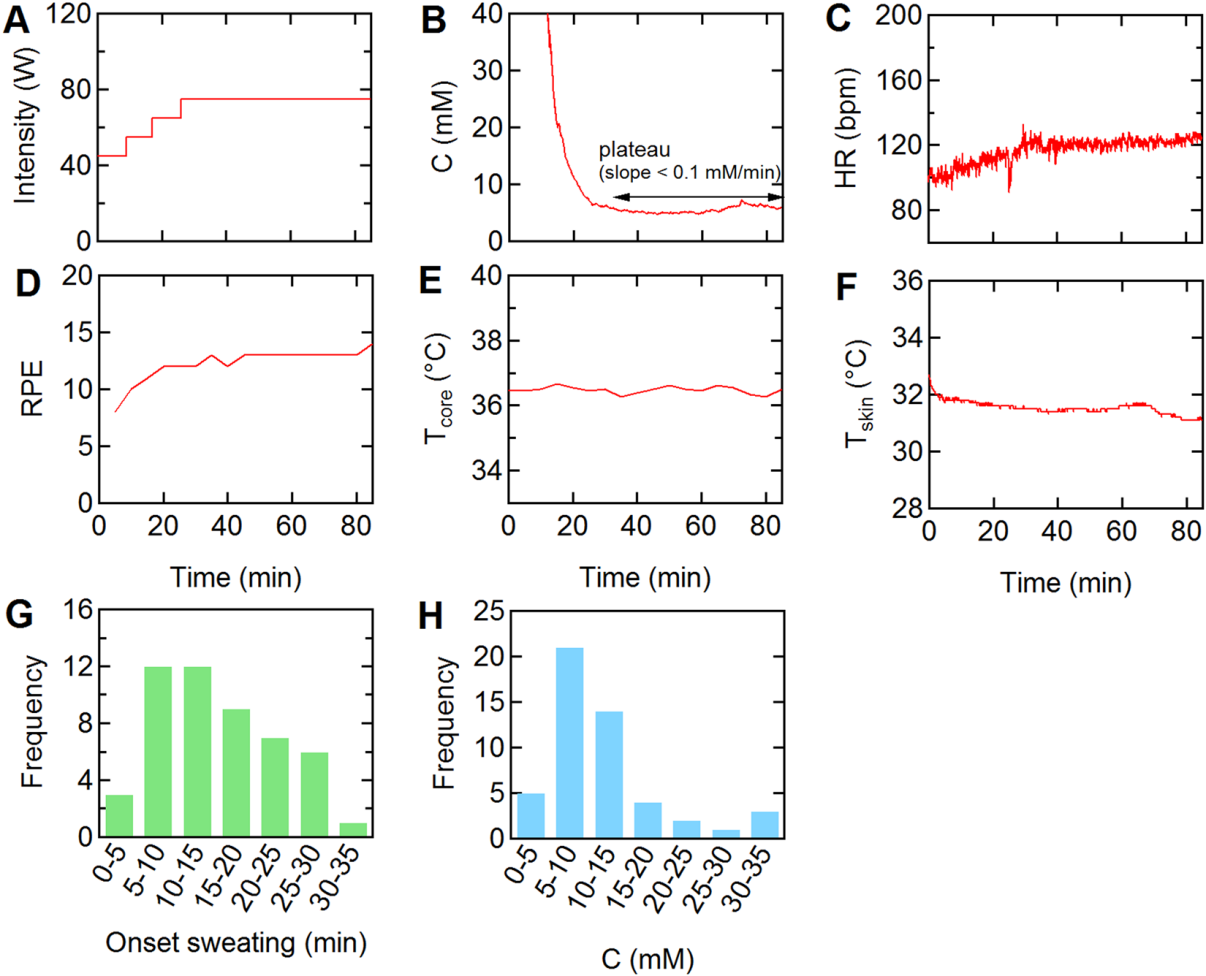
Physiological response during 60 minutes spinning at 75 W. Representative physiological measurements. (**A**) Exercise protocol: warm-up: 45 W (8 minutes), 55 W (8 minutes), 65 W (9 minutes), and 75 W (60 minutes). (**B**) Sweat chloride concentration. (**C**) Heart rate (HR). (**D**) Rated perceived exertion (RPE). (**E**) Core temperature (T_core_) using a tympanic thermometer. (**F**) Skin temperature (T_skin_) measured on the forearm. (**G**) Distribution of time at the onset of sweating. (from the beginning of the warm-up period). (**H**) Distribution of sweat chloride concentration at the first plateau (see text for details). N = 50.

The exercise protocol and the results from a typical trial are shown in Fig. 3A–F. Within a few minutes of the onset of sweating, typically at the end of the warm-up period (25 minutes), the sweat chloride concentration reached a plateau of about 5 mM (Fig. 3B). The initial transient response before reaching the plateau is due to the high sensor impedance caused by insufficient sweat and does not reflect a high sweat chloride concentration. Here we define a plateau region as a period where the slope of the sweat profile was smaller than 0.1 mM min^−1^ for at least 10 minutes. The heart rate remained at about 120 bpm and the RPE was about 12 during the 60 minutes at 75 W (Fig. 3C,D). The core temperature and skin temperature were also constant during the trial, with values of 36.5 °C and 32 °C, respectively (Fig. 3E,F). Skin temperatures are typically 3–4 °C lower than the core temperature under thermoneutral ambient conditions^28^.

The onset of sweating, determined from the change in noise level of the measured sweat concentration, generally occurred during the warm-up period (15.1 ± 7.5 minutes; mean ± CI (95% confidence interval)) (Fig. 3G). The sweat chloride concentrations in the first plateau were in the range of 2.9–34.4 mM (11.7 ± 7.2 mM, mean ± CI) (Fig. 3H). The variation in sweat chloride concentrations could be due to variation in absorption efficiency or sweat rate between individuals. In general, the sweat chloride concentration is thought to increase with increasing sweat rate, however, previous studies have relied on sweat collection over 10–30 minutes and hence the details are not well understood^29–33^. The sweat rate is relatively low at this exercise intensity, and hence the variation is likely due to individual variation in reabsorption. The range of sweat chloride concentrations is consistent with values for healthy individuals following sweat induction by iontophoresis of ≤29 mM^34^. The methods used for calculation of the onset of sweating and plateau regions are described in detail in *Materials and Methods* and Supplementary Information (Figs. S5 and S6).

### Classification of healthy individuals based on their sweat profile

**Figure 4:**
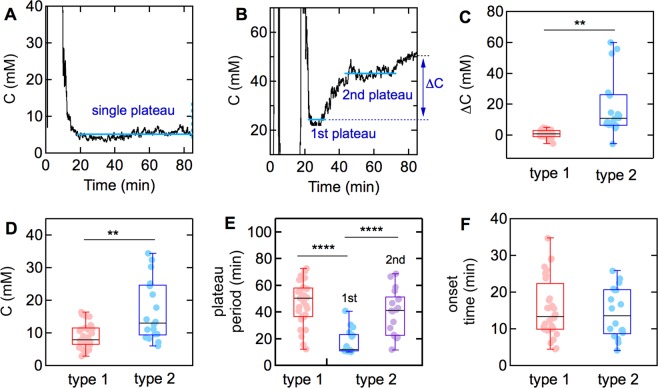
Sweat profiles of healthy subjects (N = 50) during exercise. (**A**) Representative example of a Type 1 profile with a single plateau. (**B**) Representative example of a Type 2 profile with two plateaus. (**C**–**F**) Comparison between the two groups with different sweat profiles (Type 1: single plateau, Type 2: multiple plateaus). (**C**) Changes in the sweat concentration during exercise. ΔC = C_2_ − C_1_, where C_1_ is the average chloride ion concentration for the first 10 minutes of the first plateau, C_2_ is the average chloride ion concentration from 75 to 85 minutes. (**D**) Average sweat chloride ion concentration in the first plateau. (**E**) Plateau period. (**F**) Sweat onset time. *p < 0.05, **p < 0.01, ***p < 0.001, ****p < 0.0001.

In this work, we observed two distinct responses. The Type 1 profile (N = 33) was characterized by a single-plateau (slope <0.1 mM min^−1^) (Fig. 4A), whereas the Type 2 profile showed multiple-plateaus (Fig. 4B). A single-plateau sweat profile satisfies the condition that the slope is less than 0.1 mM min^−1^ for the duration of the trial. A multiple-plateau profile had an increase in slope of greater than 0.1 mM min^−1^ after the first plateau. 14 of the 17 Type 2 profiles had two plateaus, while the remaining three profiles exhibited three plateaus or an increase but no second plateau.

To assess the differences in electrolyte loss between the two groups, we determined the concentration difference (∆C) between the average chloride ion concentration for the first 10 minutes of the first plateau and the last 10 minutes (75 to 85 minutes) of the trial. The increases in concentration (∆C) were 0.6 ± 1.8 mM (mean ± CI) for Type 1, and 17.7 ± 19 mM for Type 2 (Fig. 4C), clearly showing a difference in physiological response between the two groups (p = 0.0012). In addition to differences in ΔC, the average chloride ion concentration (at the first plateau) between the two groups was also significantly different: 9.0 ± 3.8 mM for Type 1 and 16.5 ± 9.2 mM for Type 2 (p = 0.0038) (Fig. 4D).

The length of the plateau regions was significantly longer for the Type 1 profiles (47.0 ± 7.9 minutes, mean ± CI) compared to the first plateau of the Type 2 profiles **(**15.8 ± 7.2 minutes) (p < 0.0001), but there was no difference between the lengths of the first plateau of Type 1 and the second plateau of Type 2 profiles **(**Fig. 4E**)**. In addition, the second plateau of the Type 2 profiles was significantly longer than the corresponding first plateau regions (p = 0.00002). There was no difference in the time corresponding to the onset of sweating between the two groups (Fig. 4F).

### Sweat profile and the physiological response

**Figure 5:**
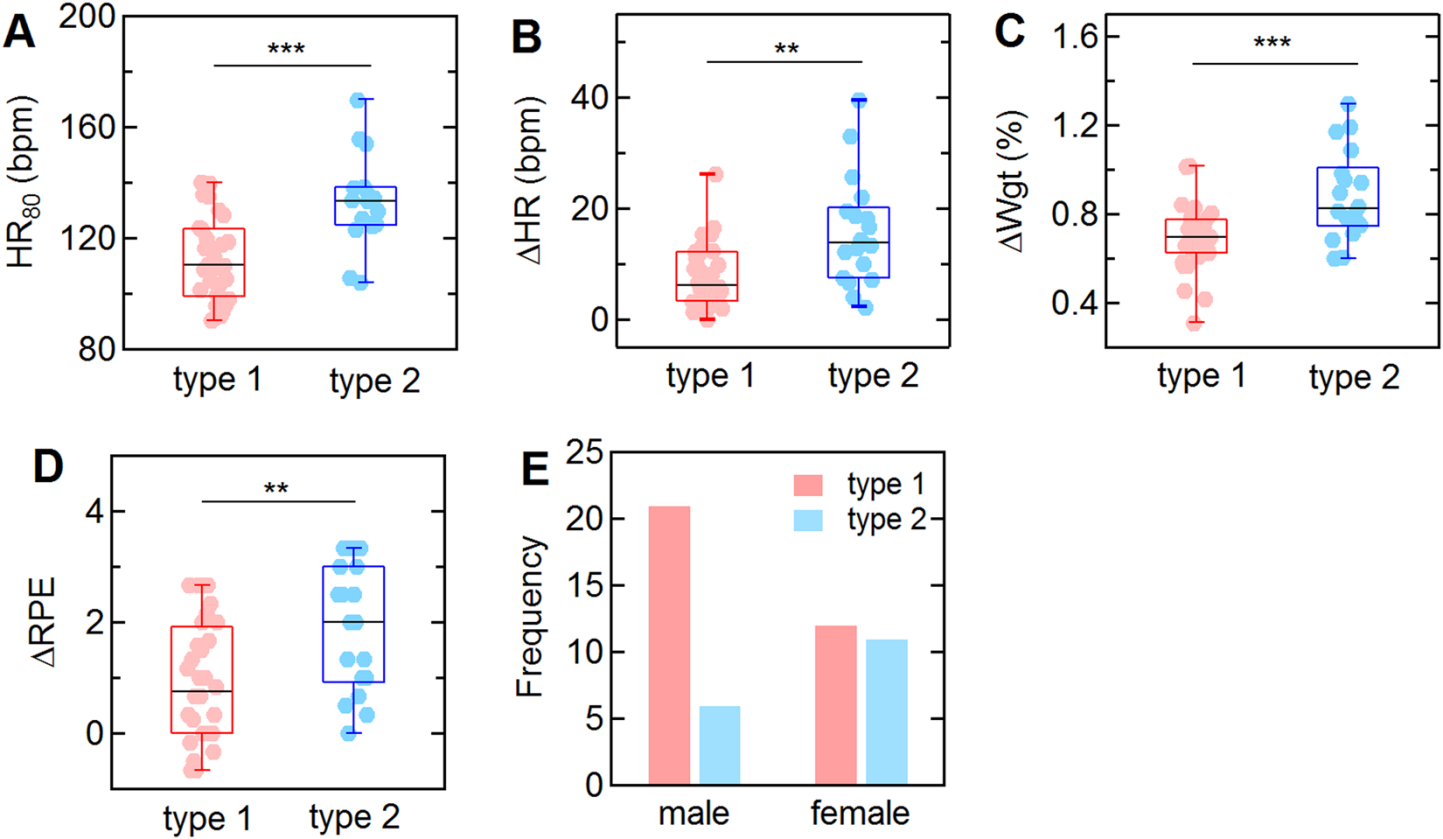
Changes in physiological parameters for individuals (N = 50) with Type 1 and Type 2 sweat profiles. (**A**) Heart rate averaged for the last 10 minutes (from 75 to 85 minutes) of the trial (HR_80_). (**B**) Change in heart rate (ΔHR). (**C**) Change in body weight (ΔWgt). (**D**) Change in Rated Perceived Exertion (ΔRPE). (**E**) Gender distribution. Changes in HR were determined from ΔX = X_2_ − X_1_, where X_1_ is the average value for the first 10 minutes of the first plateau, and X_2_ is the average value from 75 to 85 minutes. ∆Wgt was determined from the weight change at the end of the trial normalized to the weight at the beginning of the trial. ∆ RPE were obtained from the difference in the average values recorded during the first 10 minutes and last 10 minutes at 75 W. *p < 0.05, **p < 0.01, ***p < 0.001.

To assess the relationship between the sweat profile and physiological factors, we analyzed the average heart rate for the last 10 minutes (from 75 to 85 minutes) of the trial (HR_80_), which is the maximum heart rate for all subjects, along with changes in heart rate (ΔHR), RPE (ΔRPE), core (ΔT_core_) and skin (ΔT_skin_) temperatures, and body weight (ΔWgt) (Fig. 5). Individuals with Type 2 profiles had HR_80_ values of 132.8 ± 7.8 bpm during the trial, which is significantly higher than the value of 112.4 ± 5.4 bpm for Type 1 profiles (p = 0.00007) (Fig. 5A). Individuals classified with Type 2 profiles had significantly higher values of ΔHR, ΔWgt, and ΔRPE compared to those with Type 1 profiles. For example, individuals with Type 2 profiles had a change in heart rate (ΔHR) of 15.7 ± 4.5 bpm (mean ± CI), compared to 8.0 ± 2.0 bpm for Type 1 profiles (p = 0.0053) (Fig. 5B). Individuals with Type 2 profiles had a weight change of 0.88 ± 0.10%, compared to 0.69 ± 0.05% for Type 1 profiles (p = 0.0003). ΔRPE values for individuals with Type 1 and 2 profiles were 0.9 ± 0.4 and 1.9 ± 0.5, respectively (p = 0.0051) (Fig. 5C,D). The difference in core temperature was significantly larger for Type 2 profiles, but was not significant if the outlier was excluded. There was no significant difference in changes in skin temperature. While there was no gender difference in Type 2 individuals, there were significantly fewer females amongst the Type 1 individuals (Fig. 5F).

The physiological responses during exercise are interrelated. To assess the quantitative relationships among the sweat profile and other physiological parameters, principle component analysis (PCA) was performed with 11 variables (ΔC, ΔHR, ΔRPE, ΔT_core_, ΔT_skin_, ΔWgt, time for onset of sweating, age, exercise frequency, and BMI). The results from this analysis also show the strong correlation among ΔC, ΔWgt, and ΔHR (Table S1 in Supplementary Information). The eigen value of the first principle component is 22.3%, and the coefficients of ΔWgt, ΔC and ΔHR were 0.80, 0.65, and 0.77, respectively. Pearson correlation coefficients between ΔC and ΔHR, ΔC and ΔWgt, and ΔHR and ΔWgt were 0.48, 0.37 and 0.42, respectively (Fig. S7 in Supplementary Information).

### Sweat profiles and exercise intensity

**Figure 6:**
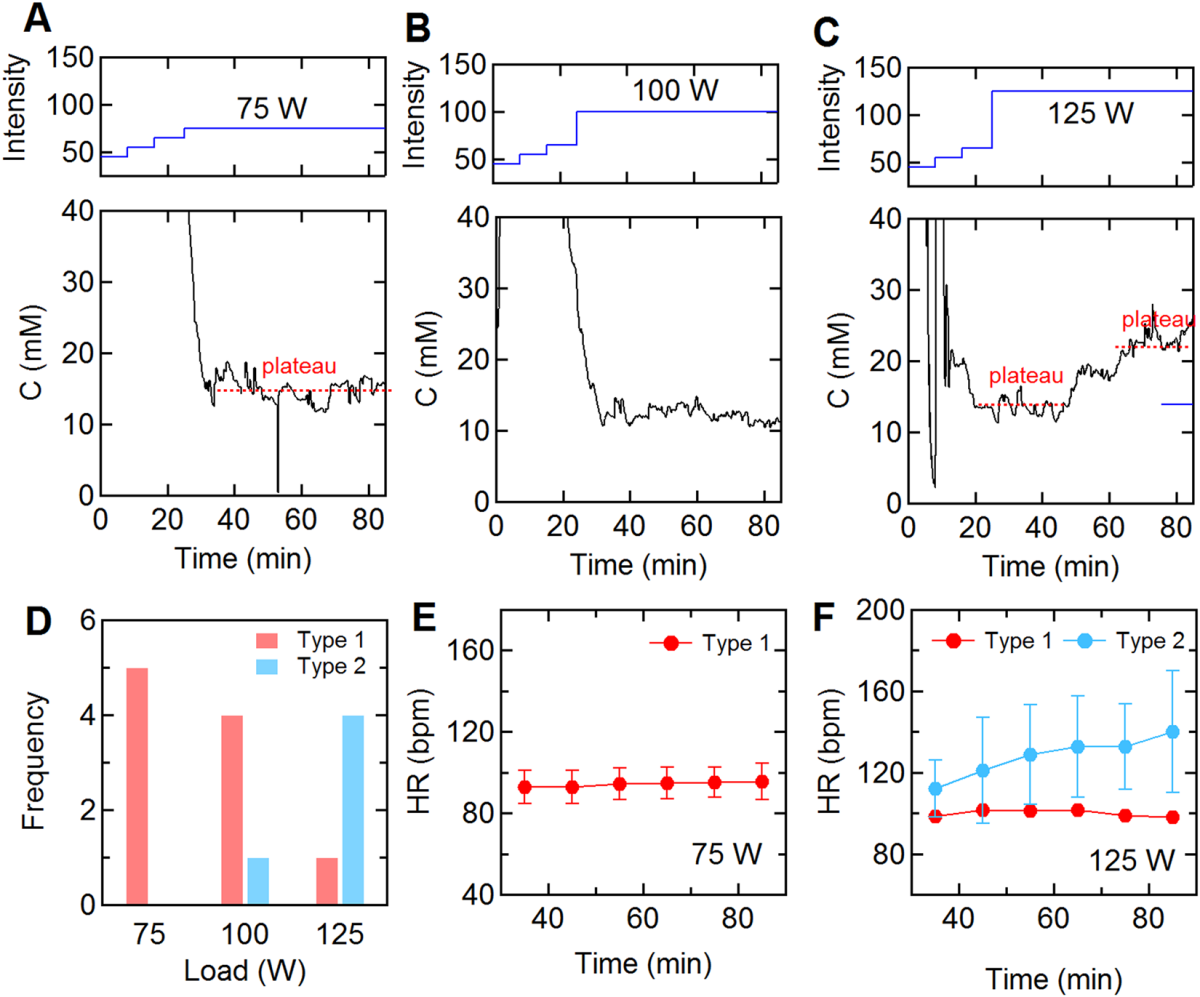
Sweat profiles and exercise intensity. (**A**–**C**) Sweat profiles for one subject in trials at exercise intensities of (**A**) 75 W, (**B**) 100 W, and (**C**) 125 W. (**D**) Dependence of sweat profile on exercise intensity. (**E**,**F**) Heart rate during (**E**) 75 W and (**F**) 125 W trials.

Since individuals with Type 2 profiles have higher HR_80_, ΔHR, and ΔRPE values, we can assume that these individuals required a higher level of effort to maintain a power output of 75 W. To assess the influence of level of effort on sweat profile, five subjects with Type 1 sweat profiles performed two additional trials at 100 and 125 W. Figure 6A–C show representative sweat profiles for one subject at the three different exercise intensities. At 75 and 100 W the sweat profiles are Type 1 with a constant sweat chloride during the trial (Fig. 6A,B), however, at 125 W the profile becomes Type 2 (Fig. 6C). During the three trails, the sweat concentrations at the plateau (75 W and 100 W) and the first plateau (125 W) were almost identical (75 W = 14.5 mM, 100 W = 12.3 mM, 125 W = 14.17 mM), but the concentration value at the second plateau during the 125 W trial increased to 23.3 mM. As the exercise intensity increased, the number of Type 2 profiles also increased (Fig. 6D). While all subjects had Type 1sweat profiles in the 75 W trials, 4 of 5 had Type 2 profiles during the 125 W trials. The change in heart rate for the four subjects with Type 2 profiles at 125 W was larger than the subject with a Type 1 profile, and increased over time during the trial (Fig. 6E,F).

### Prediction model for fluid loss during exertion

Weight change associated with fluid loss is an important metric for fatigue but is difficult to measure in real time. Since the sweat losses in this study were relatively small (<1.5% body weight), they would have no practical physiological impact^35^. However, having demonstrated that the sweat chloride profiles are related to physiological parameters (Fig. 4 and Table S1 in Supplementary Information), we next created a prediction model to classify subjects according to their weight loss during exercise. For classification we set the weight loss cut-off to the median value: 0.74% bodyweight (group 1: ΔWgt <0.74%, group 2: ΔWgt >0.74%). We employed a random forest method^36^ using ΔC, ΔHR, ΔT_core_, ΔRPE, onset time, exercise frequency, BMI, and gender as independent variables.

**Figure 7:**
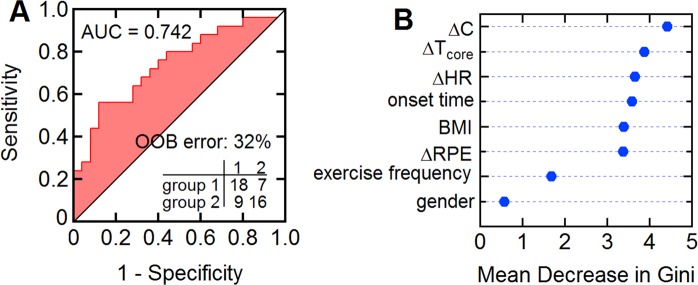
Predictive model to classify dehydration status. (**A**) OOB (out-of-bag) error, ROC (receiver operating characteristic) curve, and AUC (area under ROC curve). Sensitivity: probability that the prediction result will be group 1 when ΔWgt <0.74%. Specificity: probability that the prediction result will be group 2 when ΔWgt >0.74%. (**B**) Mean decrease in Gini coefficient (a higher value indicates more important variable).

Two metrics commonly used to evaluate the performance of random forest models are the out-of-bag (OOB) error and the AUC (area under the ROC curve, 1: perfect classification, 0.5: no better than random classification). The OOB error, an estimation of the true prediction error, was 32% (group 1: 28% (7/25), group 2: 36% (9/25)) and the AUC value was 0.742 (Fig. 7A). Interestingly, the sweat chloride concentration was the most effective variable contributing to the classification (Fig. 7B). Although a few previous studies have reported that hydration status can be classified or estimated by monitoring physiological parameters or by analyzing biofluids, these studies used *post hoc* laboratory-based analytical measurements^37–39^. Here we show that chloride ion concentration and other physiological parameters recorded using wearable devices can be used to predict fluid loss due to sweating.

## Conclusions

Wearable sensors provide new opportunities for monitoring an individual’s health state. While most wearable sensors measure physiological parameters such as heart rate, activity, and skin temperature, the development of wearable sensors for biological analytes has been more challenging. Sensors that measure the electrolyte concentration in sweat can provide new insight into time-dependent changes in health state relevant to human performance, heat exertion, and electrolyte imbalance disorders. In this work, sweat profiles were recorded at a constant moderate exercise load (N = 65). The 65 wearable sensors used in this study had a slope of −53.6 ± 3.5 mV/decade and ay-intercept of 174.4 ± 12.9 mV in the measured calibration curves. Here we show that the real-time chloride ion concentration in sweat can reflect each individual’s physiological response during exercise. Using sweat profile measured by the wearable sensor, we could classify individuals into two groups with significant different changes in heart rate, RPE, and weight losses during 1 hour of exercise at 75 W of intensity. Finally, we showed that sweat profiles along with other physiological signals can be used to predict fluid loss with out of bag (OOB) error and AUC values were 32% and 0.74, respectively. These results suggest that sweat sensors could provide real-time feedback on a range of parameters associated with exercise and exertion and enable data-driven fluid intake schedules.

## Materials and Methods

### Sweat sensor

The wearable sweat chloride sensor used in this work consists of two Ag/AgCl electrodes and a salt bridge (Fig. S2A–D in Supplementary Information). Prior to each trial, all sensors were calibrated using 10, 50, and 100 mM NaCl solution at room temperature (Fig. S2E in Supplementary Information). The calibration curves were then adjusted to skin temperature using the Nernst equation. The integrated salt bridge minimizes the equilibrium between the reference hydrogel and sweat sample, which enables accurate measurement of sweat chloride concentration for an extended period of more than 12 hours (Fig. S2F in Supplementary Information)^9,11^. In addition, the sensor has a response time of 2 s in the concentration range from 10 to 150 mM^11^. The mean difference between sweat chloride concentrations measured by the sweat sensor and the standard method used in cystic fibrosis clinics was 6.2 ± 9.5 mM, which is close to the arm-to-arm physiological variation of about 3 mM^10^. The correlation coefficient between the sensor and laboratory measurement was 0.97.

### On-body trials

All on-body trials were performed under a protocol approved by the institute review board (IRB) at Johns Hopkins University (IRB00122647). All experiments were performed in accordance with guidelines and regulations. All participants provided written informed consent before participation. Participants read the study participation informative document and signed the corresponding informed consent. 50 subjects participated in this study and their personal information including age, gender, height, weight, medical history and exercise habits were recorded (see Fig. S1 in Supplementary Information). Subjects did not consume food or water in the 3 hours before the start of the trial, and 5 mL per kg bodyweight of water was provided before the start of each trial. Identical T-shirts and shorts were provided to all subjects for the trial. All trials involved spinning on a stationary bike (LifeCycle 9500HR, Life Fitness). In this work, the exercise protocol was designed for all subjects to maintain prolonged spinning during the entire protocol, regardless of their gender, age and fitness level. Subjects warmed up by spinning at three graded exercise intensities for 25 minutes (45 W for 8 minutes, 55 W for 8 minutes, 65 W for 9 minutes). After the warm-up, subjects increased the exercise intensity to 75 W for 60 minutes. During the trials, the subjects did not consume any food or water. Room temperature was maintained at 25 °C.

During the trials, the sweat chloride concentration, heart rate, and core and skin temperatures were measured using various sensors, and the rated perceived exertion (RPE, Borg scale) was monitored by self-reporting (Fig. 2). The sweat chloride sensor was attached to a forearm using a commercial bandage (Tegaderm, 3 M) and the real-time output was monitored at a sampling frequency of 1 Hz by a Bluetooth wireless transceiver and a mobile app^10^. A heart rate monitor (Zephyr Bio Harness, Medtronic) and a thermocouple sensor (ThermoScientific, NeuLog) were employed to measure the heart rate and the skin temperature, respectively. RPE and core (tympanic) temperature values were recorded every 5 minutes. The weight change before and after the trial was measured without clothing and after removing all sweat from the body, using a conventional scale (HBF-514C, Omron, 0.1 kg resolution).

### Sweat profile analysis

The onset time for sweating was obtained by monitoring the change in electrical noise of the sweat sensor output, which decreased when the space between the sensor and the skin was filled with sweat. Here we define the onset time for sweating as the time when the standard deviation (SD) of the sensor signal was smaller than 50 mM over for 30 s (see Fig. S5 in Supplementary Information).

A plateau region in a measured sweat profile was defined as when the slope was smaller than 0.1 mM min^−1^ for at least 10 minutes (Fig. S6 in Supplementary Information). To find a plateau region, a sequential linear regression (least squares fit) was performed by applying a moving 10-minute time window. The time window was advanced in 1-second increments. The start of a plateau is the start of the first 10 minute period where the slope is <0.1 mM min^−1^, and the end of the plateau is the time where the slope exceeds 0.1 mM min^−1^. If a single plateau extended to the end of the trial, the profile was designated Type 1. Profiles were also designated as Type 1 if they exhibited two plateaus with less than 5 mM difference. Sweat profiles with two or three plateaus (with >5 mM difference), or a single plateau followed by an increase were designated as Type 2. A customized code to identify plateau regions was developed using MATLAB.

### Statistical analysis and machine learning

Statistical analysis was performed using R and SPSS software (IBM). After classifying the subjects into two groups based on their sweat profiles, a student’s t-test was performed to calculate the statistical significance between physiological parameters. Principle component analysis (PCA) was performed to interpret complex relations among the numerous measured physiological signals. Finally, a random forest algorithm was employed to develop a predictive model to classify the subjects according to their weight loss.

## Supplementary information

Supplementary Information
