# Supplementary material for: Two Distinct Types of Sweat Profile in Healthy Subjects While Exercising at Constant Power Output Measured by a Wearable Sweat Sensor
Source: Sci Rep. 2019 Nov 29;9:17877. doi: 10.1038/s41598-019-54202-1 (PMC6884585; doi:10.1038/s41598-019-54202-1)
Supplement: Supplementary file 1 — Supplementary Information [file 41598_2019_54202_MOESM1_ESM.docx]

**Supplementary Information**

**Two Distinct Types of Sweat Profile in Healthy Subjects While Exercising at Constant Power Output Measured by a Wearable Sweat Sensor**

Dong-Hoon Choi^a^, Grant Kitchen^a^, Ji Soo Kim^b^, Yi Li^a^, Kain Kim^a^, In cheol Jeong^a^, Jane Nguyen^a^, Kerry J. Stewart^c^, Scott L. Zeger^b^, and Peter C. Searson^*a,d^

**Figure S1. Participant information.**

**Figure S2**. **Wearable sweat chloride sensor.**

**Figure S3. Selectivity of the wearable sweat sensor**

**Figure S4. Fabrication processes of the wearable sweat sensor**

**Figure S5.** **Analysis of the onset time of sweating.**

**Figure S6**. **Analysis of plateau regions in sweat profiles.**

**Figure S7**. **Correlations between ΔC, ΔHR, and ∆Wgt.**

**Table S1.**  **Principle component analysis (PCA).**

**Participant information**

50 healthy individuals participated in this study (**Fig. S1**). The average age was 23.8 ± 6.5 (mean ± SD) and the numbers of female and male participants were 23 and 27, respectively. All participants were asked to refrain from smoking, drinking alcohol, and participating in exercise of greater intensity than their norm in the 24 hours leading up to the trial.


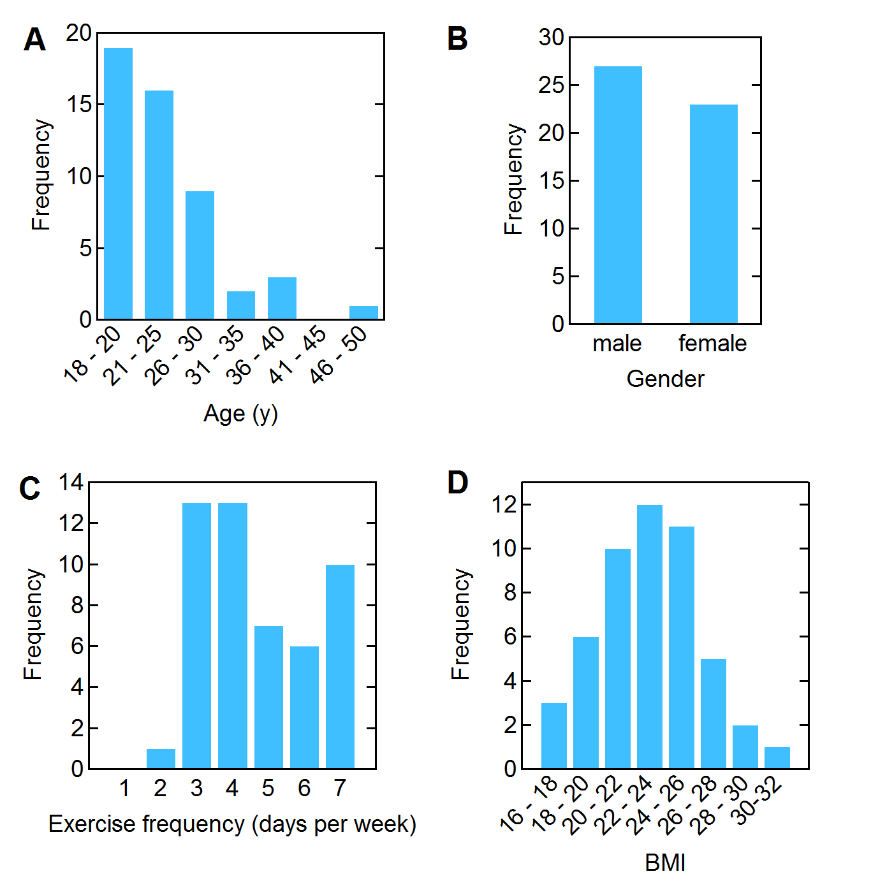


**Figure S1. Participant Information.** (A) Age. (B) Gender. (C) Exercise frequency. (D) Body mass index (BMI).

**Wearable sweat sensor**

Sweat profiles were recorded using a wearable potentiometric chloride ion sensor developed in our lab (**Fig. S2A**). The salt bridge was designed to minimize equilibration between the sweat sample and the reference solution (1 M KCl) (**Fig. S2B-D**). The sweat chloride concentration was determined from calibration curves of the sensor voltage in standard solutions. The average slope was slope of 52.8 ± 0.7 mV/decade (**Fig. S2E**) at room temperature. The concentration at skin temperature was corrected using the Nernst equation. From long term tests with 100 µL of 10 mM NaCl, the drift rate due to equilibration was 0.3 mM h^-1^ over 12 hours (**Fig. S2F**).

Calibration of the sensors was performed prior to all measurements. All devices were calibrated in the following way: (1) the working electrode was rinsed in running deionized (DI) water for 40 s, (2) 100 μL of 10 mM NaCl (Fisher Scientific) solution was placed on the working electrode of the sensor using a micropipette, (3) the sensor voltage was measured and recorded for 3 minutes, (4) steps 1 - 3 were repeated with 50 and 100 mM NaCl solutions, (5) the sensor voltage for each solution was determined by averaging the recorded voltages over last 1 min, and (6) using a linear least squares fit (V-log C), the relationship between the measured voltage and the concentration of the test solution was established. All calibrations were performed at room temperature.


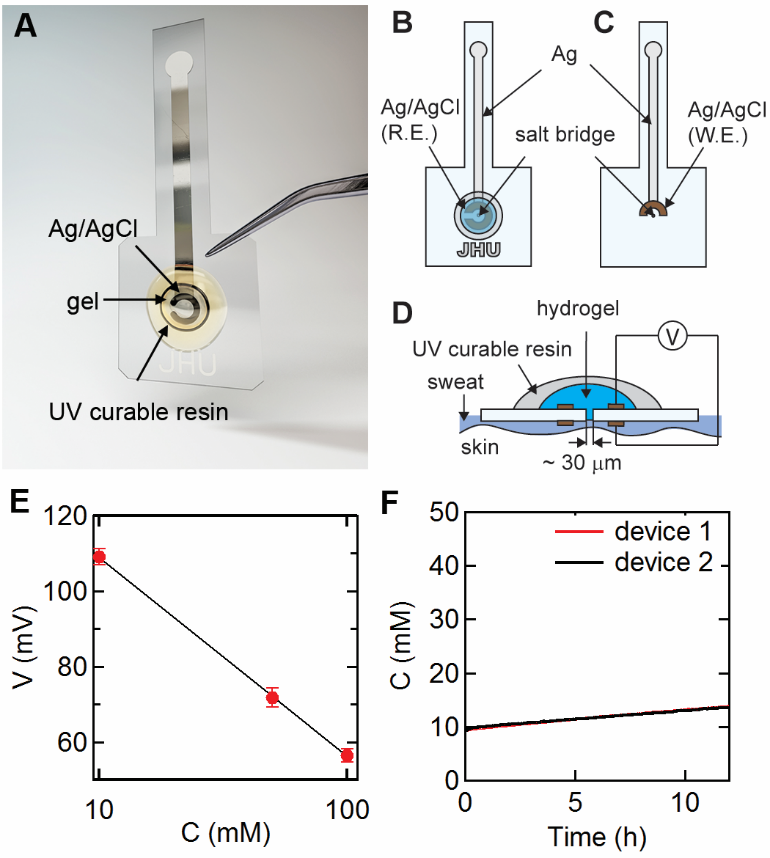


**Figure S2**. **Wearable sweat chloride sensor.**  (A) Optical image of the sensor after fabrication. Schematic illustration of the sensor: (B) top side (reference electrode), (C) bottom side (working electrode), and (D) cross-section. (E) Sensor calibration curves (N = 6). Data represent mean ± SD. (F) Measured chloride ion concentration over 12 hours in 110 μL of 10 mM NaCl.

Typically, the pH value of sweat varied from 4 to 7.5 depending on the sweat rate (reference 30 in the main manuscript). In this work, we monitored the output voltages of the sensor at different pH level (**Fig. S3A**). Even though the pH value of the test solution was abruptly changed from 4.1 to 7.5, the sensor output voltage was not changed at all. For this test, the sensor was partially immersed into pH 4.1 of 100 mM NaCl test solution so that the working electrode was completely exposed to the test solution. Then, NaOH solution (Sigma Aldrich) was added to the solution to adjust the pH value of the solution. During this, the pH of the solution was continuously monitored by a commercial pH meter (Orion 2115000). Sweat has also various cations like sodium (Na^+^, 1 – 100 mM), potassium (K^+^, 4 – 24 mM) and ammonium (NH4^+^, 0.5 – 8 mM). **Fig. 3B** shows the sensor output voltages at different sodium and potassium concentration (10, 50, 100 mM of NaCl and KCl) and there was no big change caused by the different cations.


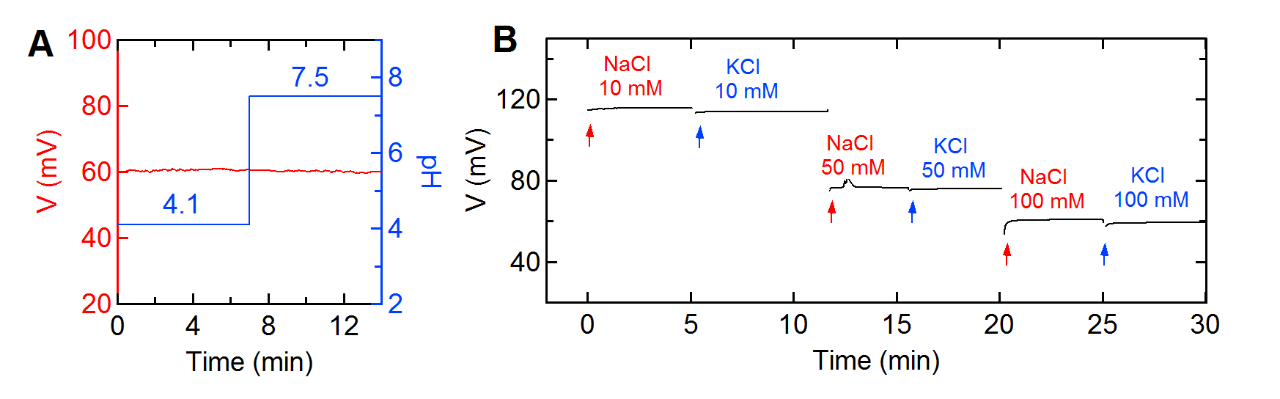


**Figure S3. Sensor selectivity.** Sensor output voltages at (A) pH 4.1 and 7.5 and (B) different cation concentrations.

The sensor was fabricated on a 125 μm thick PET (Polyethylene terephthalate, Melinex® ST) substrate (**Fig. S4**). First, the sensor substrate and the hole for the salt bridge of 30 μm in diameter were fabricated by a laser drilling process (Cobra hybrid laser, Excellon) (**Fig. S4A**). Second, a 10 nm thick Cr adhesion layer and a 300 nm thick Ag layer were deposited on both sides of the sensor substrate by e-beam evaporation (Kurt J. Lesker) and shadow masks, forming the electrodes (**Fig. S4B** and **C**). The Cr and Ag layers were deposited at deposition rates of 0.01 and 0.15 nm s^-1^, respectively, under high vacuum condition (< 5 $\times$ 10^-6^ Torr). Next, the Ag layers are turned into Ag/AgCl electrodesby treating the patterned Ag electrodes with 50 mM FeCl_3_ (Sigma-Aldrich) solution for 2.5 min (**Fig. S4D**). After that, approximately 100 μL of a 4 w/v % agarose gel (Invitrogen) containing 1 M KCl (Sigma Aldrich) reference solution was formed on the reference electrode and over the salt bridge hole (**Fig. S4E**). In this step, vacuum was applied to the salt bridge on the working electrode side to completely fill the laser drilled salt bridge hole with the gel. Finally, the gel was covered by a UV curable resin (Addison Clear Wave Coating, AC A 1450) and exposed to UV light for 4 minutes (**Fig. S5F**).


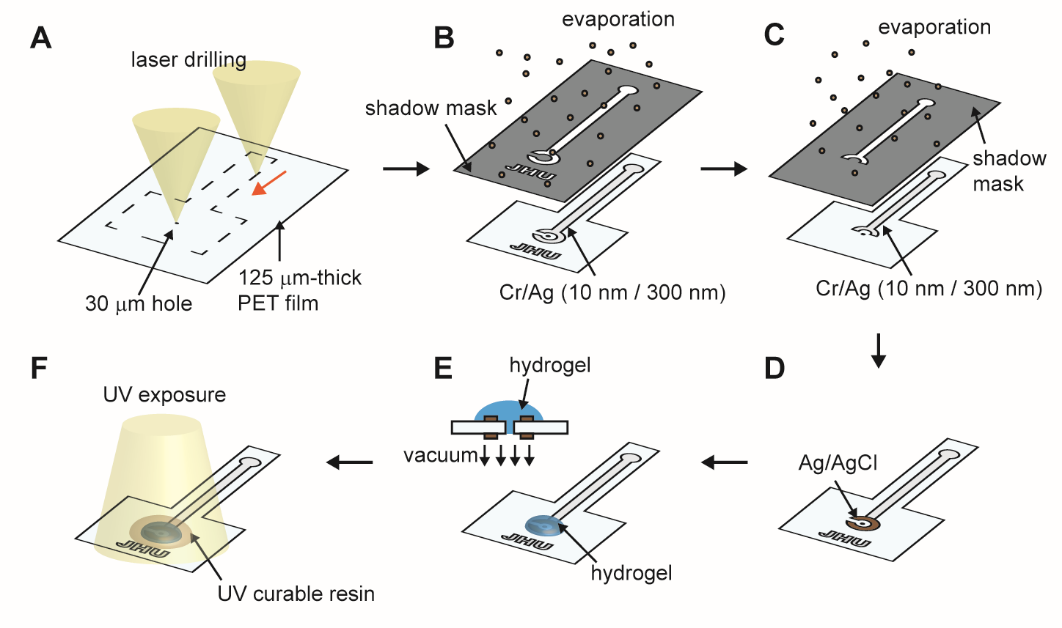


**Figure S4. Fabrication processes of the wearable sweat chloride sensor** (A) Patterning the sensor substrate and a hole for salt bridge, (B-C) e-bean evaporation of Cr/Ag (10/300 nm) layers on the top and bottom sides of the sensor substrate, (D) incubation of Ag/AgCl layer using 50 mm FeCl_3_ solution, (E) injection of a reference hydrogel on the Ag/AgCl electrode formed on the top side of the sensor substrate, and (F) sealing the reference hydrogel using a UV curable resin.

**Detection of the onset time for sweating and plateau region(s)**

*Onset time for sweating*. The onset time for sweating for each subject was determined from the change in the fluctuations of the sensor signal. Prior to the onset of sweating there are large fluctuations in the sensor output (**Fig. S5A**) due to insufficient sweat. These fluctuations decrease with time as the gap between the sensor and skin is filled with sweat. The sweat profiles were recorded at a sampling rate of 1 Hz. For each time point, we calculated the standard deviation of sensor output for the previous 30 seconds (**Fig. S5B, C**). The onset time for sweating was defined as the time when the standard deviation was smaller than 50 mM for more than 30 seconds.


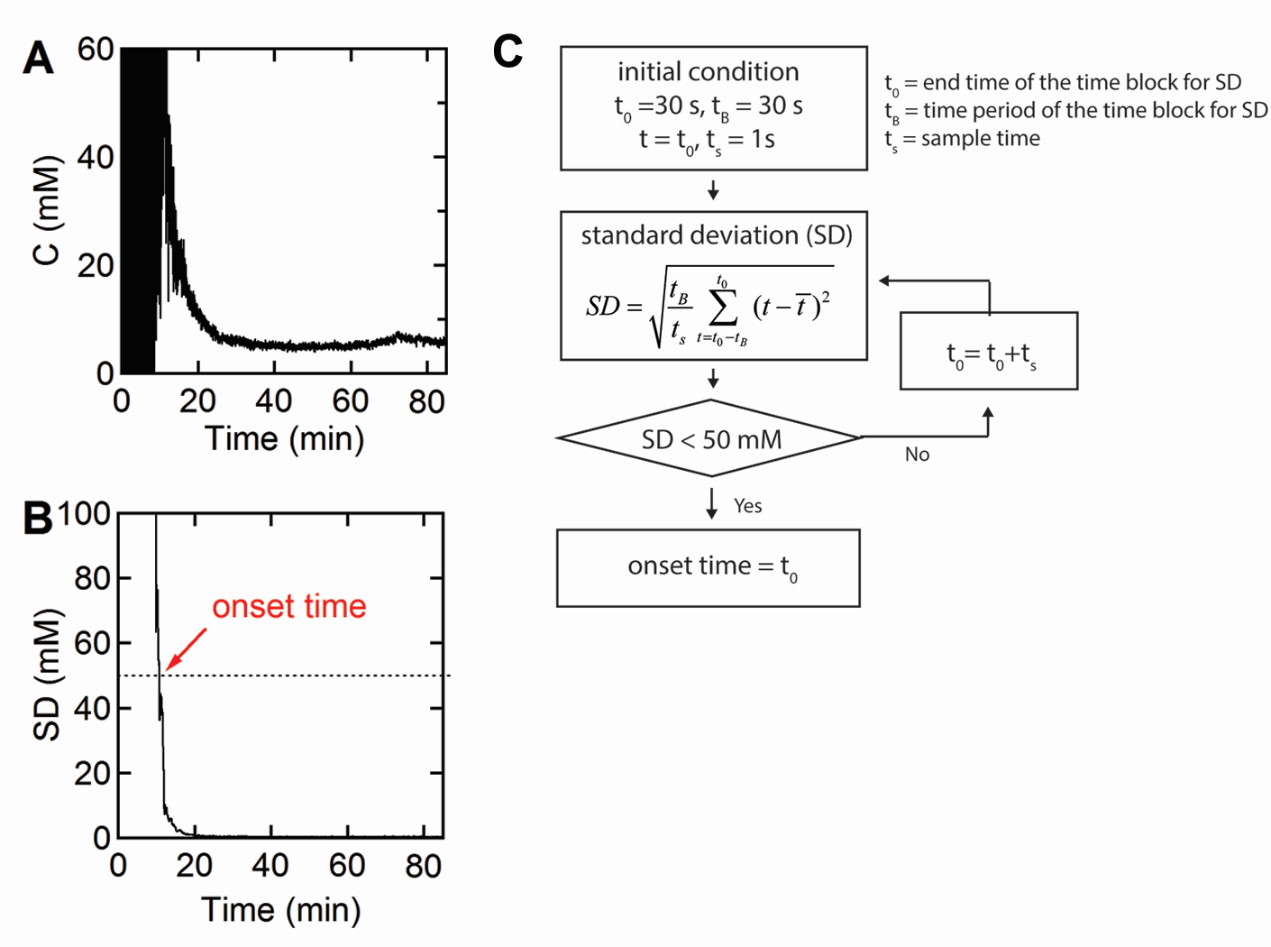


**Figure S5.** **Analysis of the onset time for sweating.** (A) Example of a measured sweat profile and (B) the corresponding signal noise. (C) Algorithm flow chart to determine the onset time for sweating.

*Plateau regions*. A plateau region in a measured sweat profile was identified from the local slope. A plateau region was defined the slope of the sweat profile was smaller than 0.1 mM min^-1^ for at least 10 minutes (**Fig. S**6). First, to detect a region with a slope < 0.1 mM min^-1^ for 10 minutes, linear regression was performed over a 10-minute moving time window. The beginning of a plateau was identified frim the first point in a 10-minute window where the slope was < 0.1 mM min^-1^. The plateau region was extended as long as the slope remained less than 0.1 mM min^-1^. If the end of the time window did not reach to the end of the trial, process was repeated until the next plateau region was detected. Profiles were then classified based on the number of the plateau regions, the end point of the first plateau region, and the concentration difference between plateau regions. Type 1 profiles were defined as profiles with a single plateau that extended to the end of trial or two plateau regions with a difference less than 5 mM. Type 2 profiles were defined as profiles with two or three plateaus (with > 5 mM difference), or a single plateau followed by an increase.


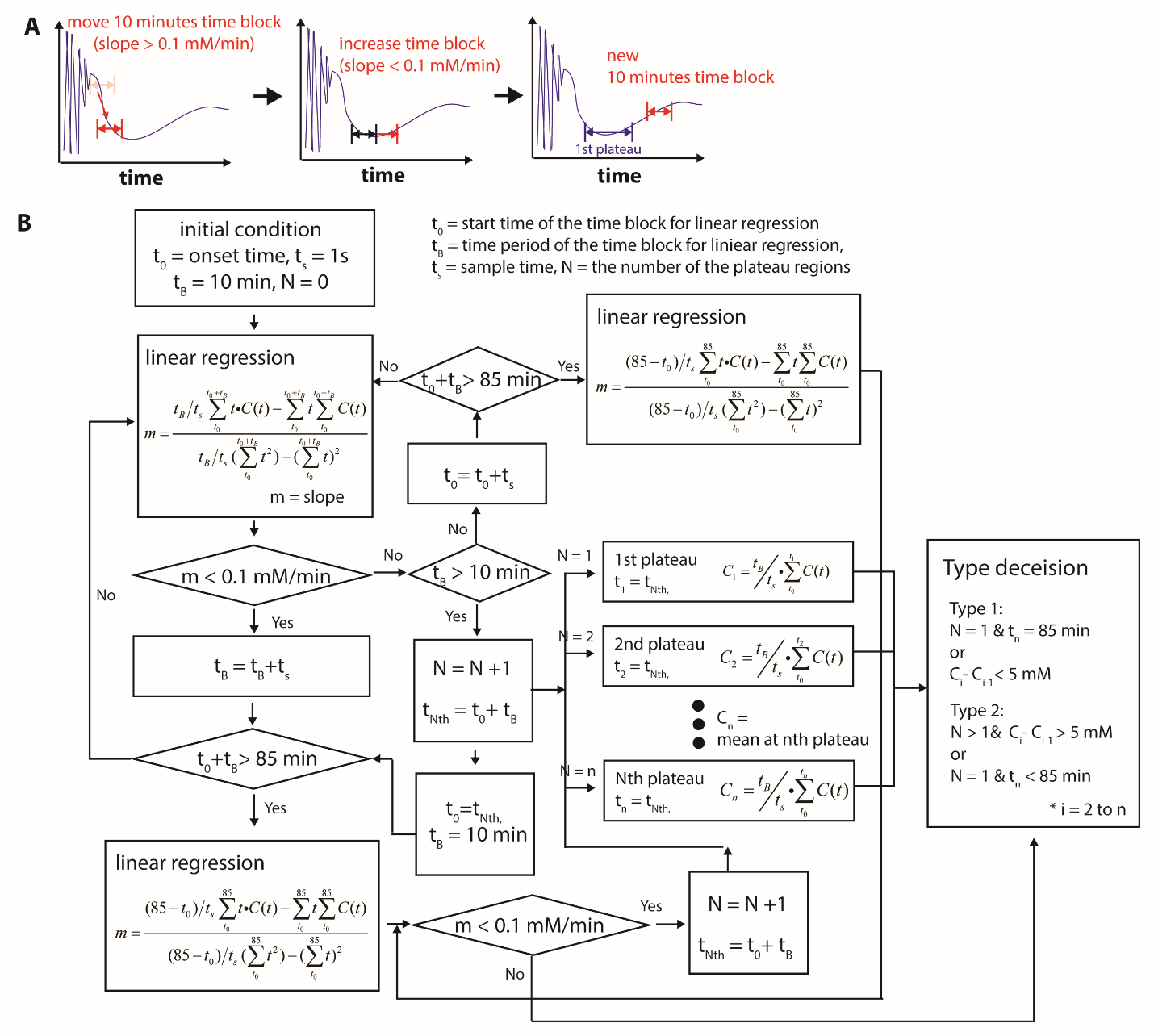


**Figure S6**. **Analysis of plateau regions in measured sweat profiles**. (A) Schematic illustration of the algorithm to detect plateau regions, and (B) flow chart.

**Correlation between change in sweat chloride concentration (∆C), change in heart rate (∆HR), and change in body weight (∆Wgt).**

The changes in sweat chloride concentration (∆C) were correlated with changes in body weight (∆Wgt) and heart rate (HR). Changes in C, HR, and body weight (Wgt) were determined from ∆X = X_2_ - X_1_, where X_1_ is the average value for the first 10 minutes of the first plateau, and X_2_ is the average value from the last 10 minutes of the trial (75 to 85 minutes).


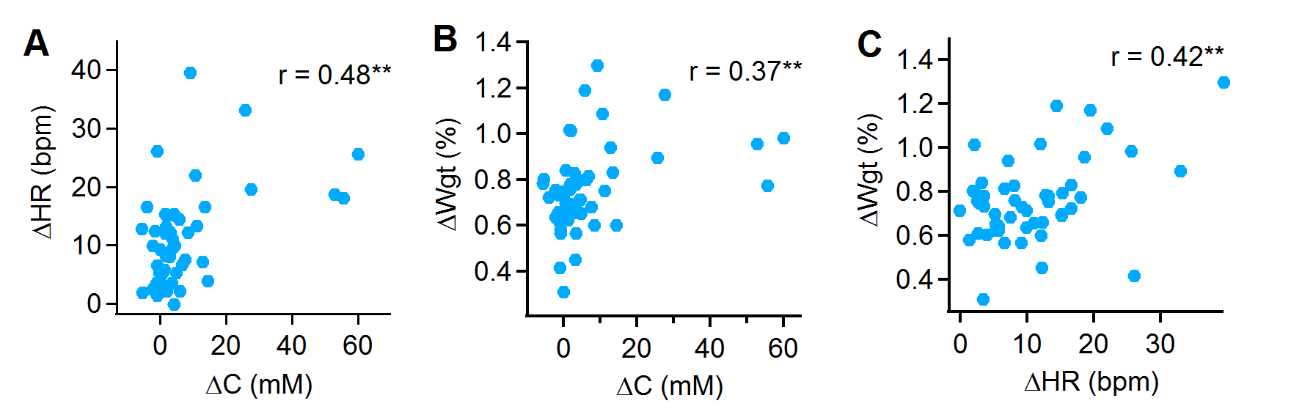


**Figure S7**. **Correlations between ΔC, ΔHR, and ∆Wgt.** (A) ΔHR and ΔC, (B) ΔWgt and ΔC, and (C) ΔWgt and ΔHR.

**Table S1.**  **Principle component analysis (PCA).** Eigen values of the principle components and the PCA coefficients for each variable. PCA analysis shows that the strongest correlations are obtained between changes in heart rate, body weight, and sweat chloride concentration (PC1). Additionally, it was observed that the subjects with larger BMI had faster time to the onset of time and exercise frequency (PC2).

|  | **PC1**  **(22.3%)** | **PC2**  **(16.6%)** | **PC3**  **(13.2%)** | **PC4**  **(12.6%)** | **PC5**  **(9%)** |
| --- | --- | --- | --- | --- | --- |
| ∆Wgt | 0.80 | 0.01 | -0.07 | -0.02 | -0.14 |
| ∆C | 0.65 | -0.20 | 0.44 | 0.27 | -0.07 |
| ∆HR | 0.77 | -0.29 | 0.09 | -0.08 | 0.27 |
| ∆T_core_ | 0.36 | -0.04 | -0.52 | -0.33 | -0.51 |
| ∆RPE | 0.40 | -0.17 | -0.56 | 0.26 | 0.50 |
| ∆T_skin_ | 0.04 | -0.25 | 0.65 | -0.38 | 0.19 |
| Onset time | -0.46 | -0.62 | 0.12 | 0.37 | -0.05 |
| Age | 0.07 | 0.18 | 0.07 | 0.83 | -0.14 |
| Exercise frequency | 0.28 | 0.67 | 0.32 | 0.13 | -0.23 |
| BMI | -0.06 | 0.77 | -0.07 | -0.06 | 0.42 |
